# COVID-19 vaccines are not associated with axonal injury in patients with multiple sclerosis

**DOI:** 10.3389/fimmu.2024.1439393

**Published:** 2024-08-22

**Authors:** Susana Sainz de la Maza, Alexander Rodero-Romero, Enric Monreal, Juan Luis Chico-García, Noelia Villarrubia, Fernando Rodríguez-Jorge, José Ignacio Fernández-Velasco, Raquel Sainz-Amo, Lucienne Costa-Frossard, Jaime Masjuan, Luisa María Villar

**Affiliations:** ^1^ Department of Neurology, Hospital Universitario Ramón y Cajal, Universidad de Alcalá, Instituto Ramón y Cajal de Investigación Sanitaria (IRYCIS), Red Española de Esclerosis Múltiple (REEM), Red de Enfermedades Inflamatorias (REI), Madrid, Spain; ^2^ Department of Immunology, Hospital Universitario Ramón y Cajal, Universidad de Alcalá, Instituto Ramón y Cajal de Investigación Sanitaria (IRYCIS), Red Española de Esclerosis Múltiple (REEM), Red de Enfermedades Inflamatorias (REI), Madrid, Spain

**Keywords:** SARS-CoV-2 immunization, neuroaxonal damage, sNfL, multiple sclerosis, COVID-19 vaccines, safety

## Abstract

**Objective:**

To evaluate the safety of COVID-19 vaccines in patients with multiple sclerosis (MS) by assessing their impact on serum neurofilament light chain (sNfL) levels as a marker of neuroaxonal damage.

**Methods:**

Single-center observational longitudinal study including patients with MS who consecutively received their initial vaccination against SARS-CoV-2 at Hospital Universitario Ramón y Cajal, following the first national immunization program in Spain. Serum samples were collected at baseline and after receiving the second dose of the vaccine. sNfL levels were quantified using the single molecule array (SIMOA) technique. Adverse events, including clinical or radiological reactivation of the disease, were recorded.

**Results:**

Fifty-two patients were included (median age, 39.7 years [range, 22.5-63.3]; 71.2% female). After SARS-CoV-2 vaccination, no increased inflammatory activity, either determined by the presence of relapses and/or new MRI lesions and/or high sNfL levels, was detected. Accordingly, there was no difference between median sNfL levels before and after vaccination (5.39 vs. 5.76 pg/ml, p=0.6). Despite this, when looking at baseline patient characteristics before vaccination, younger age associated with disease activity after vaccination (OR 0.87, 95% CI: 0.77–0.98, p=0.022). Larger studies are needed to validate these results.

**Conclusion:**

COVID-19 vaccines did not cause reactivation of disease at a clinical, radiological or molecular level, thus suggesting that they are safe in MS patients.

## 1 Introduction

Vaccine immunization of the entire population is of utmost importance for public health. The potential association of vaccination with exacerbation of multiple sclerosis (MS) disease activity has been the subject of debate for years, and the evidence remains inconclusive (1, 2).

Vaccines against Severe Acute Respiratory Syndrome CoronaVirus 2 (SARS-CoV-2) clearly reduce the proportion of people with confirmed symptomatic COronaVIrus Disease 2019 (COVID-19), and prevent severe or critical disease (3). In patients with MS, SARS-CoV-2 immunization have raised several issues. First, some disease modifying therapies (DMTs) may reduce vaccination-induced humoral immune responses, despite cell-mediated specific immune responses still provide protection (4–6). Second, cases of MS onset or reactivation temporally associated with administration of a COVID-19 vaccine have been reported (7), although a recent meta-analysis concluded that COVID-19 vaccines do not appear to increase the risk of relapse or serious adverse events (8). However, data on the impact of SARS-CoV-2 vaccination at the molecular level, which could help elucidate its safety, are still lacking.

Serum neurofilament light chain (sNfL) levels are indicative of inflammatory-driven neuroaxonal damage in patients with MS (9). Levels of sNfL increase with relapses (10), new T1 gadolinium-enhancing lesions (10, 11), and new T2 lesions (11, 12), so could serve as a sensitive molecular marker to assess the influence of COVID-19 vaccines on clinical and radiological disease activity. Whether or not SARS-CoV-2 vaccination increases sNfL levels might also have implications for future disability in MS patients, as sNfL levels have been shown to predict short-term (13, 14) and long-term (15, 16) disability worsening.

We aimed to evaluate the safety of COVID-19 vaccines in patients with MS by determining their potential impact on sNfL and relationship to disease activity.

## 2 Materials and methods

### 2.1 Study design

We performed a single-center observational longitudinal study including 52 MS patients who consecutively received a COVID-19 vaccine at Hospital Universitario Ramón y Cajal in Madrid, Spain. Inclusion criteria were: 1) diagnosis of MS according to McDonald 2017 criteria (17), and 2) receiving a full course of a SARS-CoV-2 vaccine following the first national protocol for COVID-19 vaccination (18). The study was approved by the Ethics Committee of Hospital Universitario Ramón y Cajal. Patients provided written informed consent before inclusion.

### 2.2 Data collection

At baseline, demographic characteristics, time since first MS symptoms, MS phenotype, disability according to the Expanded Disability Status Scale (EDSS) score, current DMT, and SARS-CoV-2 vaccine administered were recorded.

Patients were subsequently evaluated for 6 months after the second dose of COVID-19 vaccine to assess the safety of the immunization by evaluating the occurrence of any adverse event (AE), including acute MS relapse and worsening of previous MS symptomatology. A relapse was defined as new or recurrent neurologic symptoms lasting more than 24 hours, attributable to MS, not associated with fever or infection (17). Vaccine-related worsening of previous MS symptomatology was defined as a transient worsening of neurological function lasting less than 24 hours that occurred in the context of flu-like vaccine-induced AEs. Magnetic resonance imaging (MRI) scans were performed following the standard local protocol for DMT monitoring (i.e., an MRI at DMT initiation and every 12 months thereafter) and/or when an exacerbation of MS was suspected. Radiological activity was defined as the presence of gadolinium-enhanced activity and/or new/enlarging T2 lesions on an MRI scan.

### 2.3 Sample collection and sNfL analysis

Serum samples (5 ml) were collected before and at the earliest 28 days after vaccination, aliquoted, and stored at -80°C until studied. Neurofilament light chain values were quantified in 25 μl duplicate serum samples obtained from every patient by single molecule array (SIMOA) technique in a SR-X instrument (Quanterix, MA, USA), following manufacturer instructions. A sNfL concentration of 10 pg/ml was established as the cut-off value for defining elevated sNfL levels, based on previous studies (14, 15). A standardized score (*z*-score) for sNfL levels was used, reflecting standard deviations (SD) of absolute sNfL concentrations adjusted for age and body mass index from a normative database of healthy controls (14). A *z*-score of 1.5 was applied as the cut-off to define elevated sNfL levels, based on the literature (14, 15). Since sNfL is a nonspecific marker of axonal damage, the presence of other common causes of sNfL elevation such as head trauma, polyneuropathies or central nervous system (CNS) microvascular lesions were ruled out when sNfL concentrations were elevated.

### 2.4 COVID-19 vaccination strategy

Patients were immunized following the national protocol “Vaccination strategy against COVID-19 in Spain” (18), detailed below. In January 2021, large dependents and socio-healthcare personnel were vaccinated with one of the two mRNA vaccines available at that time: BNT162b2 (Pfizer, New York, NY, USA/BioNTech, Mainz, Germany) and mRNA-1273 (Moderna, Cambridge, MA, USA). Protocol updates were periodically published, and in February 2021 active collectives with an essential function for the Society (i.e., police, teachers) were immunized with the viral vector vaccine AZD1222 (AstraZeneca, Cambridge, UK). As of March 2021, patients with high-risk conditions were progressively included in the subsequent updates of the protocol. In May 2021 vaccination of MS patients on treatment with immunosuppressive DMTs was initiated. The hospital provided immunization with mRNA-1273 (Moderna, Cambridge, MA, USA) to all patients who met this criterion and had not been vaccinated with other vaccine because they belonged to one of the previous groups.

### 2.5 Statistical analysis

Analyses were performed using the GraphPad Prism 9.5 software (GraphPad Prism Inc, La Jolla, CA). The primary safety variable evaluated was vaccine-associated changes in sNfL. The secondary variables were vaccine-related adverse events and occurrence of clinical and/or radiological MS activity. Categorical variables were summarized using frequencies (percentages) and were analyzed with the χ2 test. Continuous variables were reported as median [range] and were analyzed with Wilcoxon rank-sum test. Logistic regression was performed to assess the effect of patient baseline characteristics on the risk of developing post-vaccination disease activity. Evidence of disease activity in the 6 months after to vaccination was included as a dependent variable, and baseline sNfL levels as an independent one. Sex, age at vaccination, type of DMT, and evidence of disease activity in the 6 months prior to vaccination were included as covariates. The Area Under the ROC Curve (AUC) was used to evaluated the logistic regression model performance. Two-tailed p-values < 0.05 were considered significant.

## 3 Results

### 3.1 Patients

Fifty-two patients were prospectively included in the study. Baseline demographic and clinical characteristics are shown in **Table 1**. Fifty patients (96.2%) were treated with DMTs at the time of COVID-19 immunization: one with platform therapies, 14 with oral DMTs, and 35 with monoclonal antibodies. Of these, eleven patients had started or switched their DMTs during the six months prior to vaccination. Most patients (93.3%) were vaccinated with the mRNA-1273 vaccine in the hospital. The remaining patients received other SARS-CoV-2 vaccines, following the national protocol for vaccination against COVID-19 (18) mentioned above: two (healthcare workers) the BNT162b2 vaccine, and other two (teachers) the AZD1222 vaccine. Twelve patients (23.1%) had had clinical and/or radiological evidence of disease activity in the 6 months prior to vaccination. Nine patients (17.3%) had elevated sNfL concentrations at baseline.

**Table 1:** Demographic and clinical characteristics.

Characteristics	Total (*n*=52)
Age, median [range] (years)	39.7 [22.5–63.3]
Females, n (%)	37 (71.2)
Time since first MS symptoms, median [range] (years)	8.0 [0.2–33.2]
MS phenotype, n (%)	
Relapsing-remitting	38 (73.1)
Secondary progressive	10 (19.2)
Primary progressive	4 (7.7)
EDSS score, median [range]	2.3 [1.0–7.0]
DMT, n (%)	
None	2 (3.8)
Platform therapies	1 (1.9)
Oral therapies	
Cladribine	9 (17.3)
Dimethyl fumarate	1 (1.9)
Fingolimod	4 (7.7)
Monoclonal antibodies	
Alemtuzumab	11 (21.2)
Natalizumab	2 (3.8)
Ocrelizumab	13 (25.0)
Rituximab	9 (17.3)
SARS-CoV-2 vaccine, n (%)	
AZD1222 (AstraZeneca)	2 (3.8)
BNT162b2 (Pfizer/BioNTech)	2 (3.8)
mRNA-1273 (Moderna)	48 (93.3)
Patients with baseline disease activity, n (%)	
Relapses	2 (3.8)
MRI activity	8 (15.4)
Relapses and MRI activity	2 (3.8)
Baseline sNfL	
sNfL concentrations, median [range] (pg/mL)	5.39 [1.74–16.67]
Patients with sNfL>10 pg/mL, n (%)	9 (17.3)
Patients with sNfL *z*-score > 1.5, n (%)	7 (13.5)

EDSS, Expanded Disability Status Scale; DMT, disease modifying therapy; MRI, magnetic resonance imaging; MS, multiple sclerosis; sNfL, serum neurofilament light chain.

**Table 2** details time-related data regarding monoclonal antibodies and induction therapies that could influence vaccination outcomes, such as time on treatment or time since last drug administration.

**Table 2:** Induction therapies and monoclonal antibodies time-related variables.

DMT	Treatment duration, median [range] (years)	Time since last infusion, median [range] (months)
Cladribine	0.51 [0.21-1.8]	3.97 [1.47-7.87]
Alemtuzumab	4.27 [0.58-5.74]	33.53 [6.97-55.9]
Natalizumab	0.18 [0.16-0.21]	
Ocrelizumab	1.46 [0.41-4.22]	3.73 [2.26-4.63]
Rituximab	1.76 [0.42-4.15]	6.07 [4.73-6.83]

DMT, disease modifying therapy.

### 3.2 Adverse events after SARS-CoV-2 vaccination

Thirty-four out of the fifty-two patients (65.4%) presented an adverse event (AE) after the first and/or the second dose of a COVID-19 vaccine. The most frequent AEs were injection site reactions (pain, redness), flu-like symptoms (fever, joint and muscle pain, malaise), fatigue and headache. In addition, two patients (3.8%) reported a temporary worsening of neurological function after first vaccination dose lasting less than 24 hours. In all cases, the AEs were mild to moderate, and hospitalization was not required. There were no cases of severe anaphylaxis or life-threatening AEs.

### 3.3 Disease activity after SARS-CoV-2 vaccination

**Figure 1:**
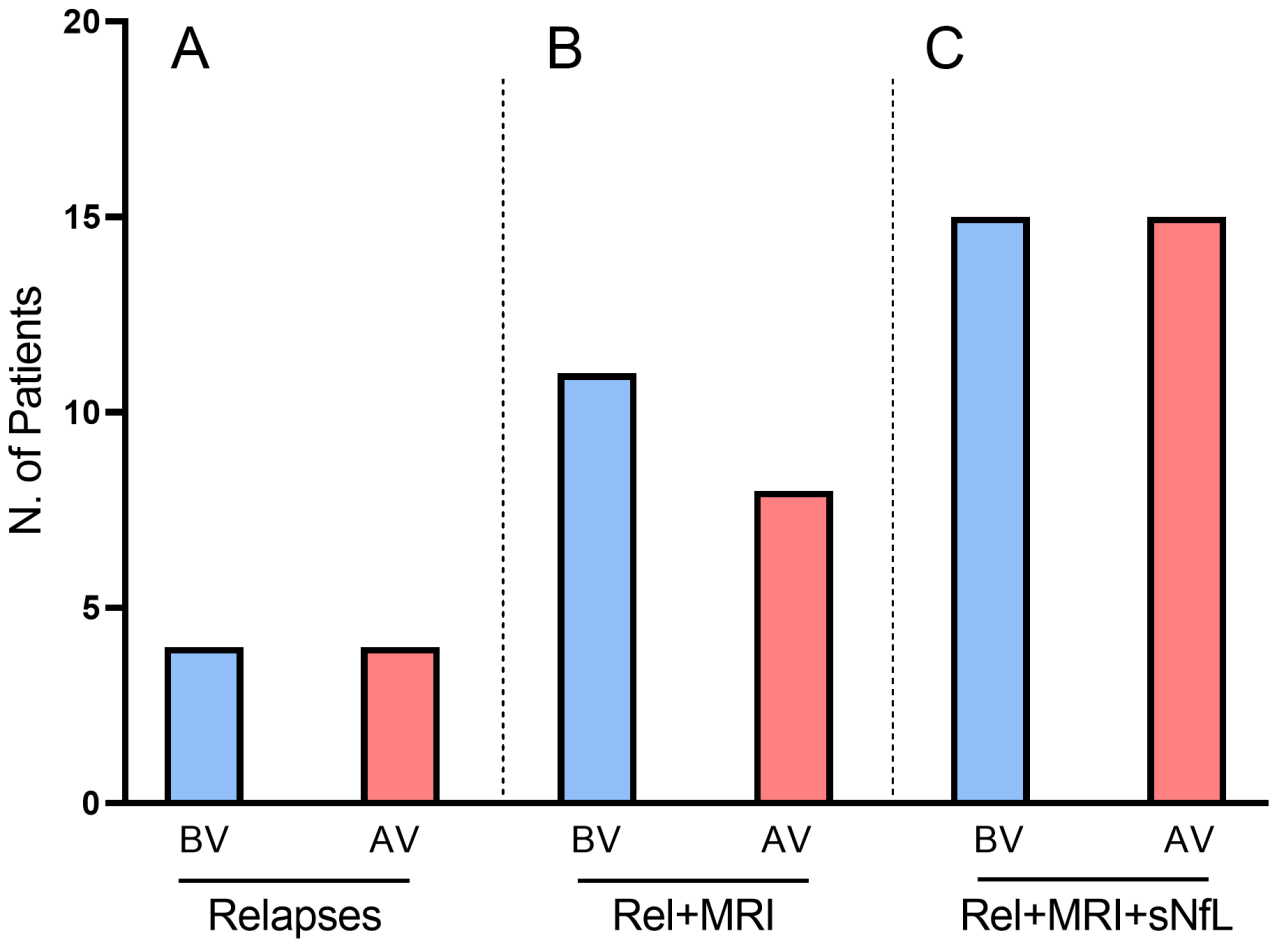
Disease activity before and after SARS-CoV-2 vaccination measured by the presence of **(A)** relapses, **(B)** relapses and/or MRI activity, and **(C)** relapses and/or MRI activity and/or elevation of sNfL levels. None of the comparisons between measures before and after vaccination were statistically significant. AV: after vaccination; BV: before vaccination; MRI: magnetic resonance imaging; Rel: relapses; sNfL serum neurofilament light chain.

Four patients (7.69%) suffered a MS relapse within the six-month observation period after SARS-CoV-2 vaccination. The mean (± SD) time from the second dose of a COVID-19 vaccine to the relapse was 89.7 (± 66.4) days. The earliest relapse occurred 37 days after the second dose of the vaccine. When we compared the percentage of patients with post-vaccination relapse with the percentage of patients with pre-vaccination relapse, we found that they were exactly the same (7.69% vs. 7.69%, p>0.5; **Figure 1A**).

Twenty-nine of the fifty-two patients (55.8%) had a brain MRI in the six-month prior to vaccination. The median time between MRI and first dose of vaccine was 134 [6-197] days. Ten of these twenty-nine patients (34.5%) showed radiological disease activity. During the six months after vaccination, twenty-seven of the fifty-two patients (51.9%) were subjected to a brain MRI study. The median time between second dose of vaccine and MRI was 127 [32-201] days. Eight of these twenty-seven patients (29.6%) exhibited radiological disease activity, which was not significantly different from the percentage of patients with radiological activity before vaccination (29.6% vs. 34.5%, p>0.5).

When we considered relapses and/or radiological activity, the percentage of patients showing disease activity did not significantly differed after vaccination (23.1% vs. 15.4%, p>0.5. **Figure 1B**).

### 3.4 Factors associated with post-vaccination disease activity

Patients with post-vaccination clinical and/or radiological exacerbation were younger than patients with no evidence of disease activity (30.8 vs. 41.9 years, p=0.003). We further analyzed whether DMTs may have influenced in disease activity. Four of the five (80%) patients with induction therapies who had an incomplete treatment course showed post-vaccination disease activity, compared to 2/15 (13.3%) patients who had already completed the treatment schedule at the time of vaccination (p=0.003). Consequently, we performed a multivariable logistic regression model. Younger age at the time of vaccination (OR 0.87, 95% CI: 0.77–0.98, p=0.022) was the only factor that increased the risk of MS exacerbation after vaccination. Other baseline characteristics did not influence this risk (**Table 3**).

**Table 3:** Multivariable logistic regression models testing the risk of post-vaccination disease activity.

Variable	OR (95% CI)	p-value
Sex (Female)	0.73 (0.09–6.10)	0.775
Age at vaccination	0.87 (0.77-0.98)	0.022
Pre-vaccination disease activity	6.73 (0.48-93.6)	0.156
DMT	0.83 (0.14-5.03)	0.845
z-score of baseline sNfL	0.71 (0.35-1.42)	0.339

CI, confidence interval; DMT, disease modifying therapy; OR, odds ratio; sNfL, serum neurofilament light chain.

### 3.5 Change in sNfL levels after SARS-CoV-2 vaccination

**Figure 2:**
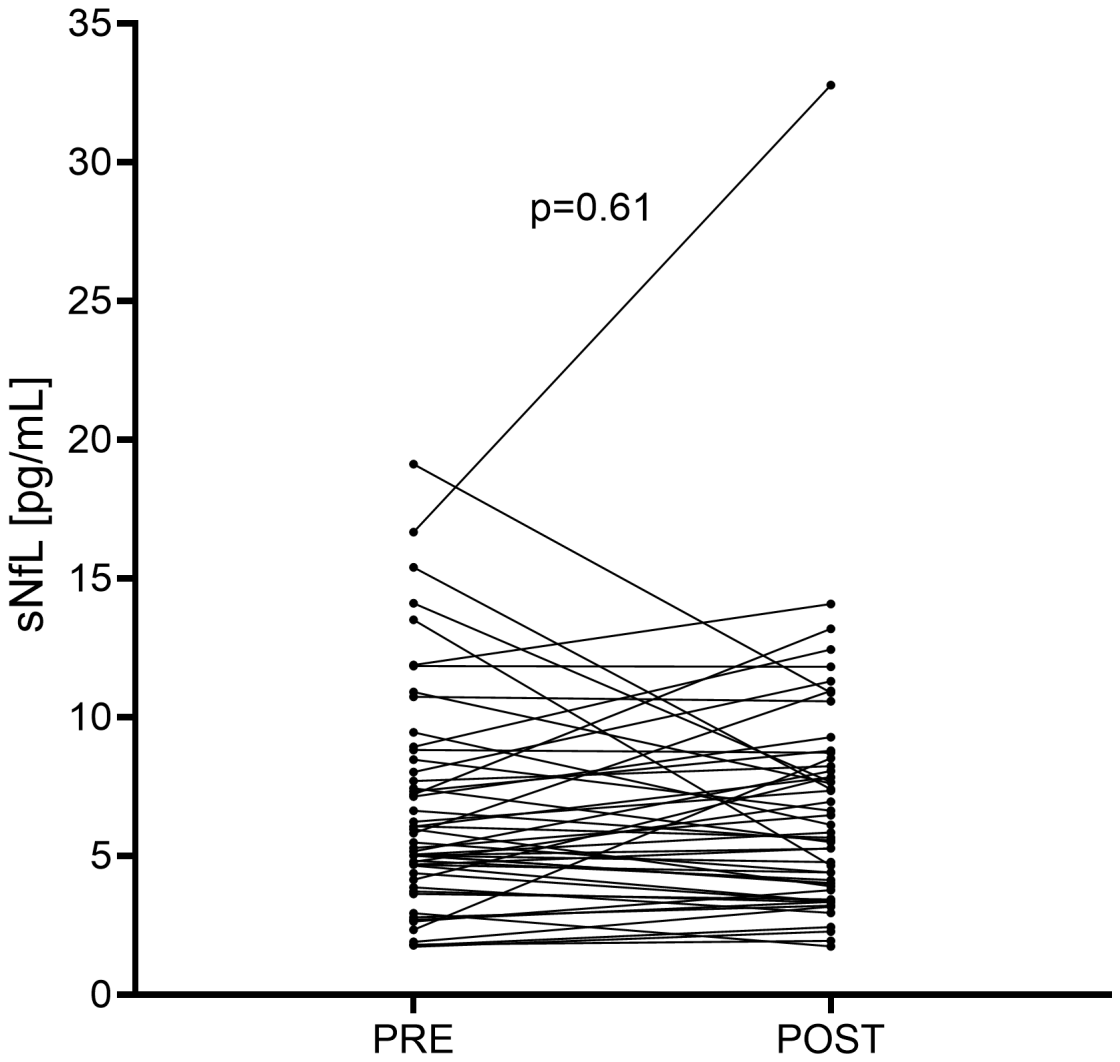
Changes in serum neurofilament light chain (sNfL) levels after SARS-CoV-2 vaccination. Each line connecting a “PRE” point to a “POST” point represents a patient. POST: post-vaccination. PRE: pre-vaccination.

Median sNfL levels before vaccination were 5.39 [1.74-16.67] pg/ml. After a mean (± SD) time of 72.4 (± 26.5) days from first vaccine dose, median post-vaccination sNfL levels were 5.76 [1.35-32.78] pg/ml. No significant differences were found between sNfL values before and after vaccination sNfL values (**Figure 2**). We further explored the percentage differences in sNfL concentrations between baseline and follow-up in each patient and found that the median percentage of change was 2.23% [-65.5% - 89.1%], supporting that there was little variation in sNfL levels before and after vaccination. We finally analyzed the change in *z*-score before and after vaccination. Before vaccination, median *z*-score of sNfL concentrations was -0.825 [-3.72 – 2.65] while after vaccination was -0.295 [-3.16 – 2.24]. No significant difference was found between the *z*-score of the sNfL values before and after vaccination.

Nine of the fifty-two patients (17.3%) had elevated sNfL concentrations before vaccination. Of these patients, four normalized their levels after vaccination. In contrast, four of the forty-three patients (9.30%) with low baseline sNfL values experienced an increase in sNfL above 10 pg/ml after vaccination. Overall, the percentage of patients with elevated sNfL before and after vaccination was the same (17.3% vs. 17.3%, p=1.0). Alternatively, we repeated the analysis using a cut-off value of 1.5 for the sNfL *z*-score and the results were similar to those obtained with 10 pg/mL of sNfL as a cut-off. The percentage of patients with elevated sNfL *z*-score was not statistically higher before vaccination than after vaccination (13.5% vs. 9.62%, p=0.41).

When we considered relapses and/or radiological activity and/or elevated sNfL, the percentage of patients showing some evidence of disease activity was the same before and after COVID-19 vaccination (28.8% vs. 28.8%, **Figure 1C**).

## 4 Discussion

We conducted an observational study to assess safety of COVID-19 vaccines in MS patients, not only at a clinical level, but also at molecular level by comparing serum neurofilament light chain levels before and after vaccination.

Vaccination against SARS-CoV-2 plays a crucial role in public health by preventing the spread of the infection, reducing disease severity, and decreasing mortality rates (19, 20). In patients with MS, it is especially important, as some evidence suggests that COVID-19 may exacerbate MS symptoms, leading to neurological worsening and increased disability (7, 21). However, as vaccines could theoretically increase the risk of CNS autoimmune disorders through mechanisms similar to those induced by infections (22), MS patients may be reluctant to receive SARS-CoV-2 immunization. This calls for data on specific markers of neuroaxonal damage that could elucidate the real impact of COVID-19 vaccines at the molecular level.

Neurofilament light chain is a promising biomarker of neuroaxonal damage that associates with acute inflammation (9–12), correlates with treatment response (12), and predicts progression of disability worsening in MS patients (13–16). The role of sNfL to investigate the safety of COVID-19 vaccines has been under-explored. However, it could be a useful tool, as elevated sNfL levels have been shown to be a surrogate marker of axonal injury (13). Preliminary results of a recent study concluded that sNfL levels did not increase after vaccination against tetravalent influenza virus in a small cohort of 20 MS patients receiving dimethyl fumarate (23). Accordingly, our results showed that immunization against SARS-CoV-2 is not associated with an elevation of sNfL in patients with MS. This strongly suggest that vaccinated patients are not at increased risk of acute disease activity, failure of DMT or long-term disability progression.

Several studies explored the occurrence of relapses after SARS-CoV-2 vaccination in MS patients. The idea that it might lead to a MS relapse is primarily based on individual case reports or series (24), but a recent systematic review and meta-analysis of pharmacovigilance registries and observational studies with more than 14,000 MS patients concluded that COVID-19 vaccines do not appear to increase the risk of relapse (8).

A systematic review of the reported cases of CNS demyelination in association with COVID-19 vaccines revealed that most cases occurred after the first dose of the vaccine, with neurologic symptoms manifesting after a median of 9 days (25). In the literature, 28 days is considered appropriate for evaluating MS exacerbations after immunizations (23). In our study, no patients relapsed after first dose of a COVID-19 vaccine, and the earliest relapse occurred 37 days after the second dose.

Data on MRI in patients who received a COVID-19 vaccine and did not experience a relapse is scarce in literature. A multicentric observational study that found no significant difference in MRI disease activity between vaccinated and unvaccinated patients with radiologically isolated syndrome (RIS) (26), suggesting that COVID-19 vaccines are safe at a radiological level. In our study, four patients had some degree of radiological activity that was elicited after a follow-up brain MRI, but they had no clinical activity. This suggests that MRI imaging should be strongly considered when assessing post-vaccination disease activity.

We went a step further in the assessment of disease activity and measured the levels of sNfL, which is a sensitive biomarker associated with acute inflammation. We thus confirmed absence of increased clinical and/or subclinical disease activity following vaccination against SARS-CoV-2.

Predictors of relapse after SARS-CoV-2 vaccination identified in a study cohort of over 2,000 MS patients were missing immunotherapy and a shorter time from the last pre-vaccination relapse to the first vaccine dose (27). Another study cohort of 1,661 vaccinated MS patients found that younger age was associated with relapse after SARS-CoV-2 vaccination or infection (28). In our study, both younger age and having received incomplete induction therapy were associated with disease activity after immunization, although only age proved to be a true risk factor in the logistic regression model. Accordingly, treating physicians should be aware of a possible increase in MS activity following SARS-CoV-2 vaccination in younger patients and consider taking precautions. Apart from this, our study demonstrated that COVID-19 vaccines are safe in MS patients at a population level.

The main limitation of the study is the small sample size, which makes the cohort heterogeneous in terms of baseline characteristics as age or disease course. In contrast, there was little variability in the DMT received by our cohort of patients since the protocol indicated to start by vaccinating patients treated with monoclonal antibodies (18). Another limitation of our work is that the vast majority of patients received the Moderna vaccine, because it was the one administered in our Centre. However, this limitation could also become an advantage as it resulted in a more homogeneous patient sample.

In conclusion, this study demonstrates that COVID-19 vaccines are not associated with an increase of sNfL levels, further demonstrating that this vaccination does not result in disease exacerbation in the majority of patients with MS. Larger studies are needed to validate our findings.

## Data Availability

The original contributions presented in the study are included in the article/supplementary material. Further inquiries can be directed to the corresponding author.
